# Early results and complication rate of the LapiCotton procedure in the treatment of medial longitudinal arch collapse: a prospective cohort study

**DOI:** 10.1007/s00402-022-04399-0

**Published:** 2022-03-21

**Authors:** Cesar de Cesar Netto, Amanda Ehret, Jennifer Walt, Rogerio Marcio Kajimura Chinelati, Kevin Dibbern, Kepler Alencar Mendes de Carvalho, Tutku Erim Tazegul, Matthieu Lalevee, Nacime Salomão Barbachan Mansur

**Affiliations:** 1 Department of Orthopedics and Rehabilitation, Carver College of Medicine, University of Iowa (UIOWA), 200 Hawkins Drive, Iowa City, IA 52242 USA; 2 Department of Radiology, Carver College of Medicine, University of Iowa (UIOWA), Iowa City, USA; 3 Department of Orthopedic Surgery, Rouen University Hospital, Rouen, France; 4 Department of Orthopedics and Traumatology, Escola Paulista de Medicina, UNIFESP, São Paulo, Brazil

**Keywords:** Flatfoot, PCFD, Hallux valgus, Midfoot arthritis, Weight-bearing computed tomography, WBCT, Lapidus, Cotton, LapiCotton

## Abstract

**Introduction:**

Instability/collapse of the medial column has been associated with many conditions, particularly progressive collapsing foot deformity (PCFD), hallux valgus (HV), and midfoot arthritis (MA). Restoration of first ray length and sagittal plane alignment to restore the foot tripod is essential when treating these deformities. This study aimed to assess early results, healing, and complication rate of a distraction dorsal opening plantarflexion wedge allograft first tarsometatarsal joint fusion (LapiCotton Procedure) in patients with collapse/instability of the medial column.

**Methods:**

In this prospective cohort study, we included PCFD, HV, and MA patients that underwent a LapiCotton procedure. Fusion site healing was defined by > 50% bone bridging in both interfaces between allograft wedge and host bone using weight-bearing computed tomography (WBCT) after 3 months. First ray collapse radiographic correction and minor and major complications (deep dehiscence, deep infection, and reoperation) were assessed.

**Results:**

A total of 22 patients (22 feet) were included (11 PCFD, 6 MA, and 5 of HV patients). Mean follow-up was 5.9 months (range 3–12) and median allograft size was 8 mm (range 5–19 mm). Bone healing was observed in 91% of cases. Two minor complications (9%, both superficial dehiscence) and one major complication (4.5%, deep infection) were observed. Statistically significant improvement of the sagittal plane talus-first metatarsal angle was observed, with mean improvement of 9.4° (95% CI 6.7–12.1°; *p* < 0.0001).

**Conclusion:**

In this prospective cohort study of 22 patients treated with the LapiCotton procedure for medial longitudinal arch collapse/instability, we observed a low complication rate (9% minor, 4.5% major), high healing rate after 3 months (91%), one clinically stable radiographic non-union (4.5%) and one unstable non-union (4.5%) needing reoperation. Our results demonstrate promising initial results for LapiCotton technique in treating collapse of the medial longitudinal arch in patients with PCFD, MA and HV deformities. Long-term results are needed to confirm these promising results.

**Level of evidence:**

Level II, prospective cohort study.

## Introduction

The crucial role of the first ray and the entire medial column in instituting and preserving the position of the three-dimensional (3D) foot tripod is a well-accepted concept in the literature [1–3]. Biomechanical and structural incompetence of the first ray with instability and collapse of the medial longitudinal arch has been associated with many conditions, especially progressive collapsing foot deformity (PCFD) [4, 5], hallux valgus (HV) [6, 7], and midfoot arthritis (MA) [8–12]. Fusion of the first tarsometatarsal joint (TMT), or the modified Lapidus procedure [13], is a well-accepted and long-lasting advocated procedure that allows correction of the first metatarsal malposition in the axial, coronal and sagittal planes, and potentially reestablishes the structural stability of the medial column [14–19]. However, non-union rate of up to 8% [20] and relative shortening and dorsiflexion of the first metatarsal are inherent possible limitations and complications associated with the procedure [21–24], with an average of 4.1 mm decrease in absolute length of the first ray being reported in the literature [25, 26]. Combination of first ray shortening and dorsiflexion can lead to transfer load to the lesser metatarsals and residual mechanical incompetence of the medial column [7, 20].

A bone block first TMT joint arthrodesis is a historically described procedure used as salvage to restore the first ray length [27, 28]. Traditionally, the concept applies to sequela of midfoot trauma and revision for failed Lapidus procedures [29], aiming to restore the length of the first ray, improve the mechanical lever arm of the medial column, and decrease the load transfer to the lesser metatarsals [27, 28]. However, no emphasis on sagittal plane and dorsiflexion deformity correction has been made with this procedure.

On the other hand, the Cotton osteotomy was described several years ago with the intent to plantarflex the first ray and rebuild the “triangle of support” of the foot [3]. Through the increase of the plantar inclination of the first metatarsal with a dorsal opening wedge of the medial cuneiform, forefoot varus/supination and medial arch collapse can be corrected [3, 30]. Several authors demonstrated the procedure’s capability in improving alignment and outcomes for PCFD patients [31, 32]. Still, the effect of Cotton osteotomies in the overall stability of the first ray is still a matter of debate [33].

A surgical technique combining the mechanical advantages of a Cotton osteotomy and a modified Lapidus procedure, or LapiCotton, has been recently described by de Cesar Netto et al. to treat collapse of the medial column, by means of fusing the first TMT using a dorsal opening wedge distraction allograft [34]. The procedure would have the potential advantages of maintaining/increasing the length of the first ray and plantarflexing the medial column, restoring the mechanical competence of the first ray in the foot tripod and at the same time allowing the conventional corrections of rotational and transverse plain malalignment. However, questions regarding the effectiveness and safety of the procedure, non-union of the fusion site, overcorrection, and other complications associated with the procedure have not been solved yet. Therefore, the aim of this study was to report the early results and complication rate of the LapiCotton procedure in a prospective cohort of patients with collapse/instability of the medial column of the foot. Our hypothesis was that a low complication rate and high healing rate of the osteotomy site and a significant amount of correction of the collapse of the medial column of the foot would be observed.

## Methods

This study complied with the Health Insurance Portability and Accountability Act (HIPAA) and the Declaration of Helsinki. In addition, it obtained an Institutional Review Board approval before its start (#IRB 202012422).

### Design

In this prospective comparative cohort study, we enrolled consecutive adult patients (over 18 years old) that underwent the LapiCotton procedure for mechanical restoration of the medial column as part of the surgical treatment of PCFD, HV and MA. All patients underwent weight-bearing computed tomography (WBCT) assessment of the affected foot and ankle preoperatively, 12 weeks and 1-year postoperatively. WBCT was also performed after 6 months if there was no complete healing of the LapiCotton fusion site at the 3-month WBCT [35, 36]. Studies were performed with a cone-beam computed tomography extremity scanner (PedCAT™, CurveBeam® LLC, Warrington, PA, USA). Participants were directed to stand with the feet aiming frontward, set at shoulder width, distributing the body weight uniformly between the lower limbs, bearing weight in a physiological straight position [37]. Using dedicated software (Cubevue, CurveBeam® LLC, Warrington, PA, USA), multiplanar data were converted into sagittal, coronal, and axial plane images.

Patients were excluded if they had the diagnosis of peripheral neuropathy and/or Charcot arthropathy. Patients that underwent the procedure but did not have at least 3 months of clinical follow-up were not included.

### Subjects

**Fig. 1:**
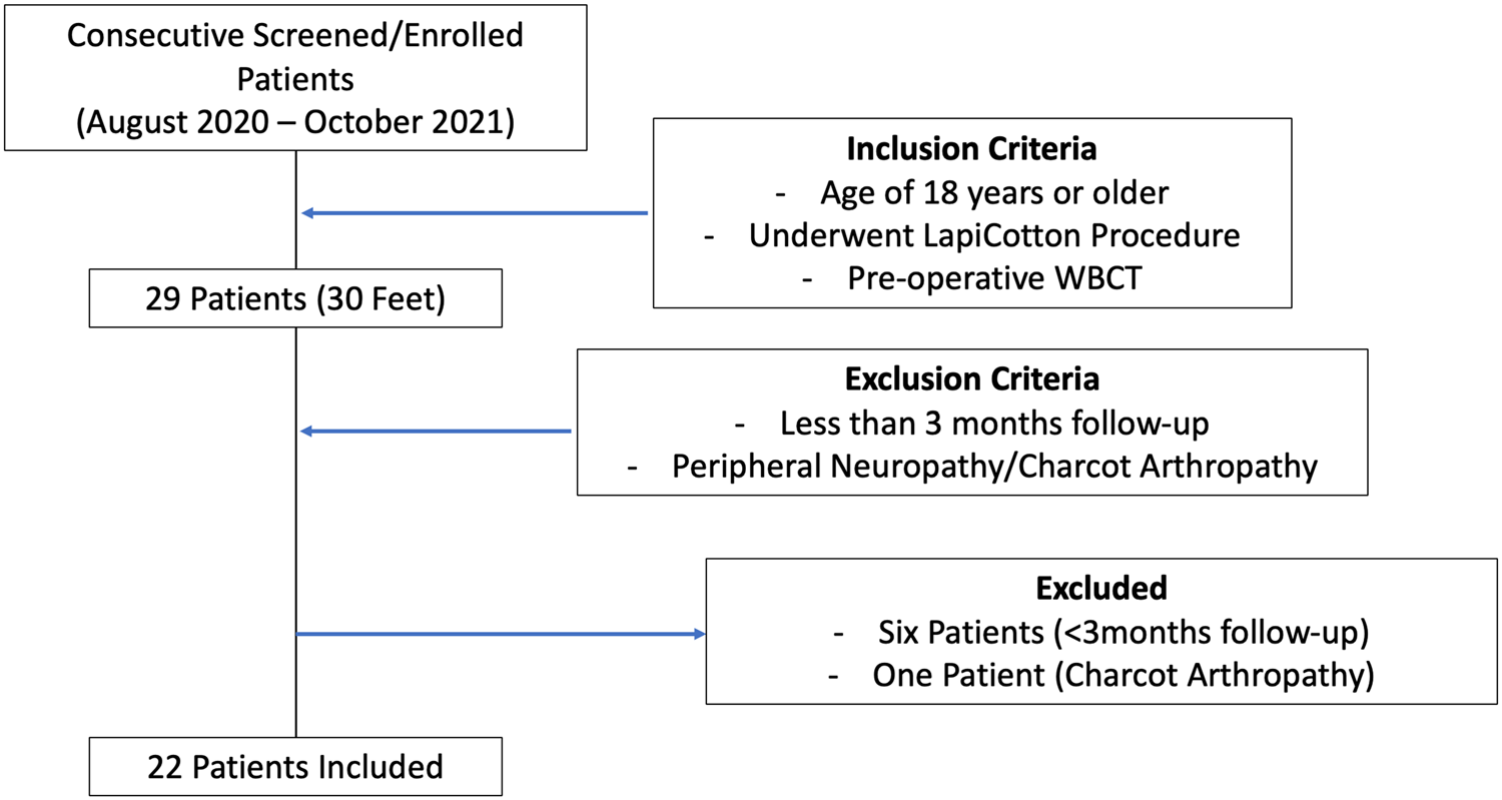
CONSORT diagram of enrolled, excluded, and included patients

A total of 29 consecutive individuals (30 feet) that underwent the LapiCotton procedure from August 2020 to October 2021 were screened and enrolled. After inclusion and exclusion criteria assessment, 22 patients (22 feet) were included in this analysis, 5 males (22.3%) and 17 females (77.3%), 12 left side (54.5%) and 10 right side (45.5%), with a mean age of 52.6 years (range, 19–75 years; SD, 14.4), and a mean body mass index (BMI) of 32.9 kg/m^2^ (95% CI, 29.2 to 36.5). A CONSORT diagram of enrolled, excluded and finally included patients is presented in Fig. 1. Of the 22 patients included, 11 patients were treated for PCFD (50%), 6 had MA (27%), and 5 had the diagnosis of HV (23%).

### Conventional surgical technique for the LapiCotton procedure

**Fig. 2:**
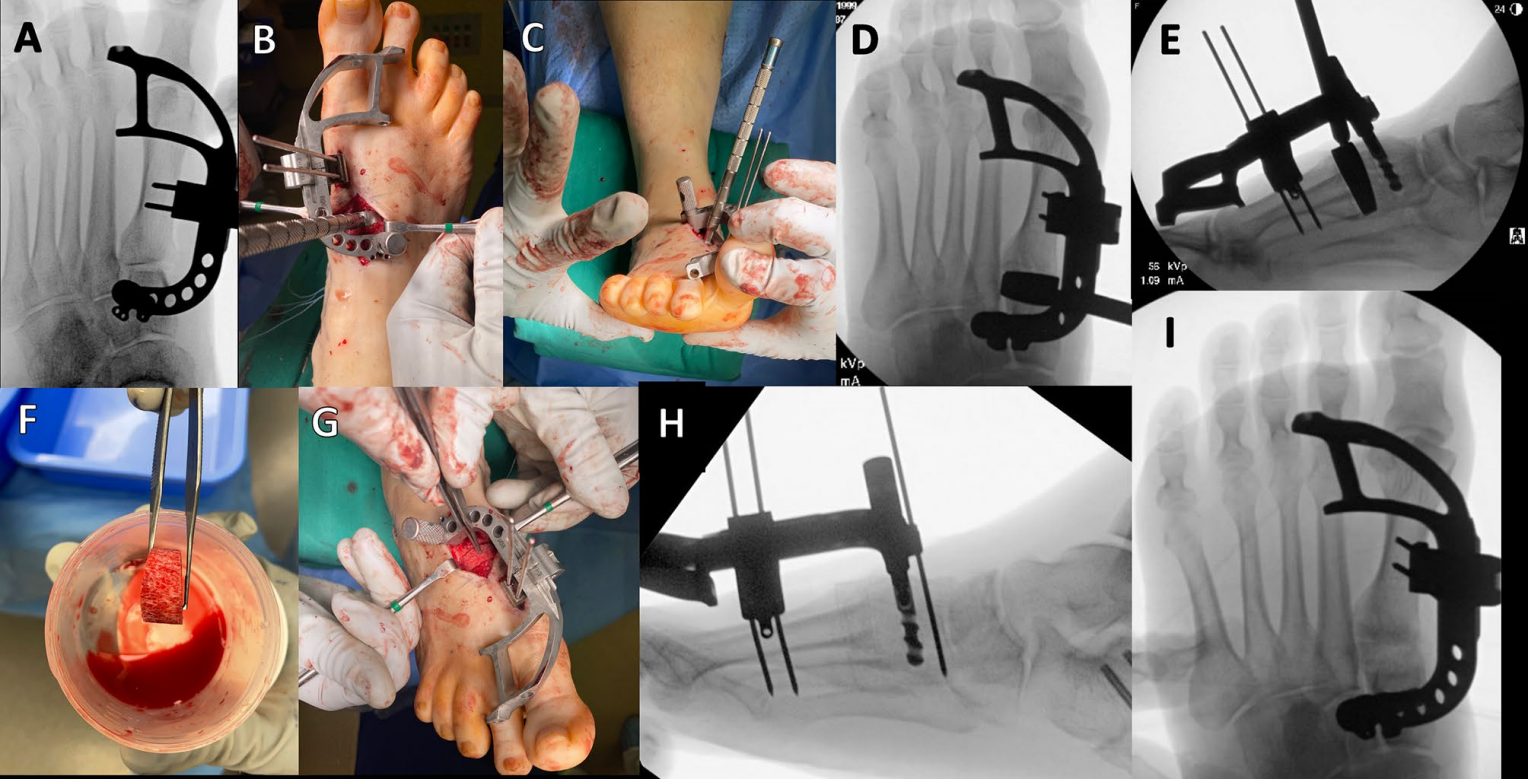
Conventional Lapicotton surgical technique in a patient with progressive collapsing foot deformity (PCFD). After the medial cuneiform post connected to the external jig is inserted into the medial cuneiform, the coronal plane position of the first metatarsal is corrected, if needed, and two K-wires are inserted in the external jig distal slots into the first metatarsal shaft, stabilizing the construct. After distraction through the jig and adequate joint preparation (**A**), the different trial sizes for the dorsal opening wedge allograft are inserted in the joint space (**B**). Sizing is checked by assessing the amount of correction of the first ray plantarflexion, assessed mainly clinically by palpating the heads of the first, fourth, and fifth metatarsals (**C**). The objective is that the first metatarsal head should be at the same level or plantarly positioned in relation to the lateral column metatarsal heads. Then, fluoroscopically, the relative length of the first and second metatarsal is evaluated (**D**). The goal is to have the length of the first metatarsal equal or slightly shorter than the second metatarsal. The relationship between the talus and first metatarsal in the sagittal plane using a lateral foot fluoroscopic view is also performed (**E**), aiming to correct the collapse of the first ray. The determined size of allograft wedge is thawed in the back table and soaked in the iliac crest bone marrow aspirate concentrate (**F**). Finally, it is inserted in the fusion site, while maintaining the distraction with the external jig (**G**). Transverse plane deformity can be performed manually by applying manual pressure distally over the medial aspect of the first metatarsal head, closing the intermetatarsal angle if needed. Compression is applied through the jig, a third K-wire is inserted through a proximal slot in the jig locking the transverse plane correction and the final position is checked under fluoroscopy (**H**, **I**). Distal post screws can be then inserted using appropriate targeted slots in the external jig, finalizing the fixation

Important surgical steps of the published conventional LapiCotton procedure are depicted in Fig. 2 [34]. The first TMT joint was then exposed with a 5–6 cm-long dorsomedial approach. A guide wire for insertion of the cuneiform post implant (Zimmer-Biomet® InCore^©^ system, Warsaw, IN) was positioned from dorsal to plantar. Once the position of the guide wire was confirmed, the tunnel for insertion of the cuneiform implant was drilled. The single-size implant was then attached to the external jig and manually inserted into the medial cuneiform tunnel. Two K-wires were inserted through specific slots distally in the external jig. While maintaining multiplanar correction, an additional K-wire was inserted proximally through the jig. The external jig was then utilized to distract the first tarsometatarsal joint. Joint preparation was performed. The size trials for pre-shaped allograft Lapidus wedges (Preserve wedges^©^, Paragon28®, Denver, CO) were inserted in the fusion site. Clinically, the amount of correction was subjectively evaluated. The flexibility of the first MTP joint was also assessed to ensure that overdistraction of the first ray did not happen, limiting the range of motion of the first MTP joint. Radiographically, the amount of correction was assessed by checking the relationship between the length of the first and second metatarsal head in anteroposterior fluoroscopic view, making sure that the first ray was not overlengthened, aiming for a first metatarsal with the same length or slightly shorter than the second metatarsal. Once the size of the wedge was decided, the appropriately sized allograft wedge was soaked in the iliac crest bone marrow aspirate concentrate and introduced into the fusion site. Compression of the fusion site was then performed through the external jig. Two post screws were then inserted distally using the targeted guide through the external jig. Additional procedures were performed as needed. When a plate or a nail was used (alternatively to the post), the above steps of preparation and correction were repeated, and only the implant was changed. Patients were kept non-weight bearing for 6 weeks, followed by progressive weight bearing in a boot.

### Outcomes

Healing of the fusion site (both surfaces) was assessed by two blinded and independent fellowship-trained orthopedic foot and ankle surgeons using weight-bearing cone-beam CT at the 3-month follow-up. Disagreement between two readers was decided by a third blinded and independent reader, a fellowship-trained musculoskeletal radiologist. A percentage greater than 50% of crossing trabeculae/bone bridging along both proximal and distal surfaces between host bone and allograft was defined as a healed fusion site [38, 39]. Absence of bone bridging of more than 50% in any of the fusion site surfaces was defined as absence of healing/delayed healing. No partial healing was considered. Non-union was defined as absence of the same signs of healing at the 6-months follow-up.

Complications were assessed until the most recent follow-up. Minor complications were established by superficial dehiscence, superficial infection, and neuropraxia [40]. Superficial dehiscence was the inability to heal the soft tissue coverage until the end of the 4th week after surgery. Superficial infection was defined as the presence of local phlogistic signs or increased drainage requiring the use of oral antibiotics, and that was resolved without the need of a surgical intervention.

Major complications were defined as deep dehiscence, deep infection needing intravenous antibiotic or surgical intervention, and need for reoperation [40, 41]. The presence of persistent (more than 12 weeks) clinical complaints related to first ray length/plantarflexion overcorrection such as pain under the first metatarsal head/sesamoids, limited range of motion of the first MTP joint, and overload of the lateral column were also considered major complications. [42].

### Measurements

**Fig. 3:**
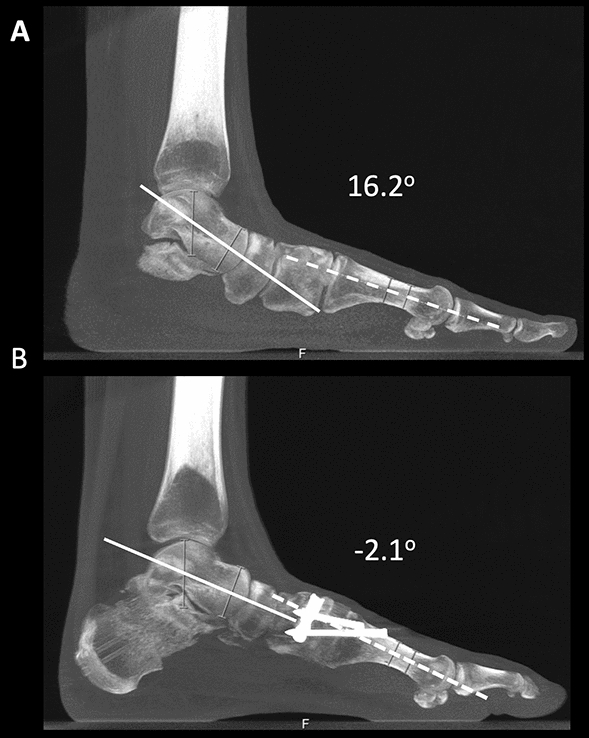
Example of preoperative (**A**) and postoperative (**B**) measurements of weight-bearing CT sagittal plane talus-first metatarsal angle (TFMA) in a progressive collapsing foot deformity patient is demonstrated. The preoperative TFMA was 16.2° of collapse of the medial longitudinal arch (plantar vertex). It was corrected postoperatively to 2.1° of dorsal apex (negative value), with a total correction of 18.3°

Two independent and blinded observers, both fellowship-trained orthopedic foot and ankle surgeons, measured the talus-first metatarsal angle (TFMA) in the sagittal plane of WBCT images, preoperative and postoperatively [43–47]. It was considered positive if it had a plantar vertex (collapse) and negative if it had a dorsal vertex. Example of preoperative and postoperative TFMA measurements in one of the treated patients is depicted in Fig. 3.

### Statistical analysis

Continuous variables were assessed for normality by the Shapiro–Wilk test and reported using mean, median, range and mode values. The 95% confidence interval was reported when applicable. The reliability between two readers for the talus-first metatarsal angle was assessed by the intraclass correlation coefficient (ICC), considering bias and interactions. ICC higher than 0.8 was considered excellent agreement [48]. Preoperative and postoperative TFMA was compared by paired *T* test or paired Wilcoxon test. The JMP Pro 15 Software was used for statistical analysis.

## Results

**Fig. 4:**
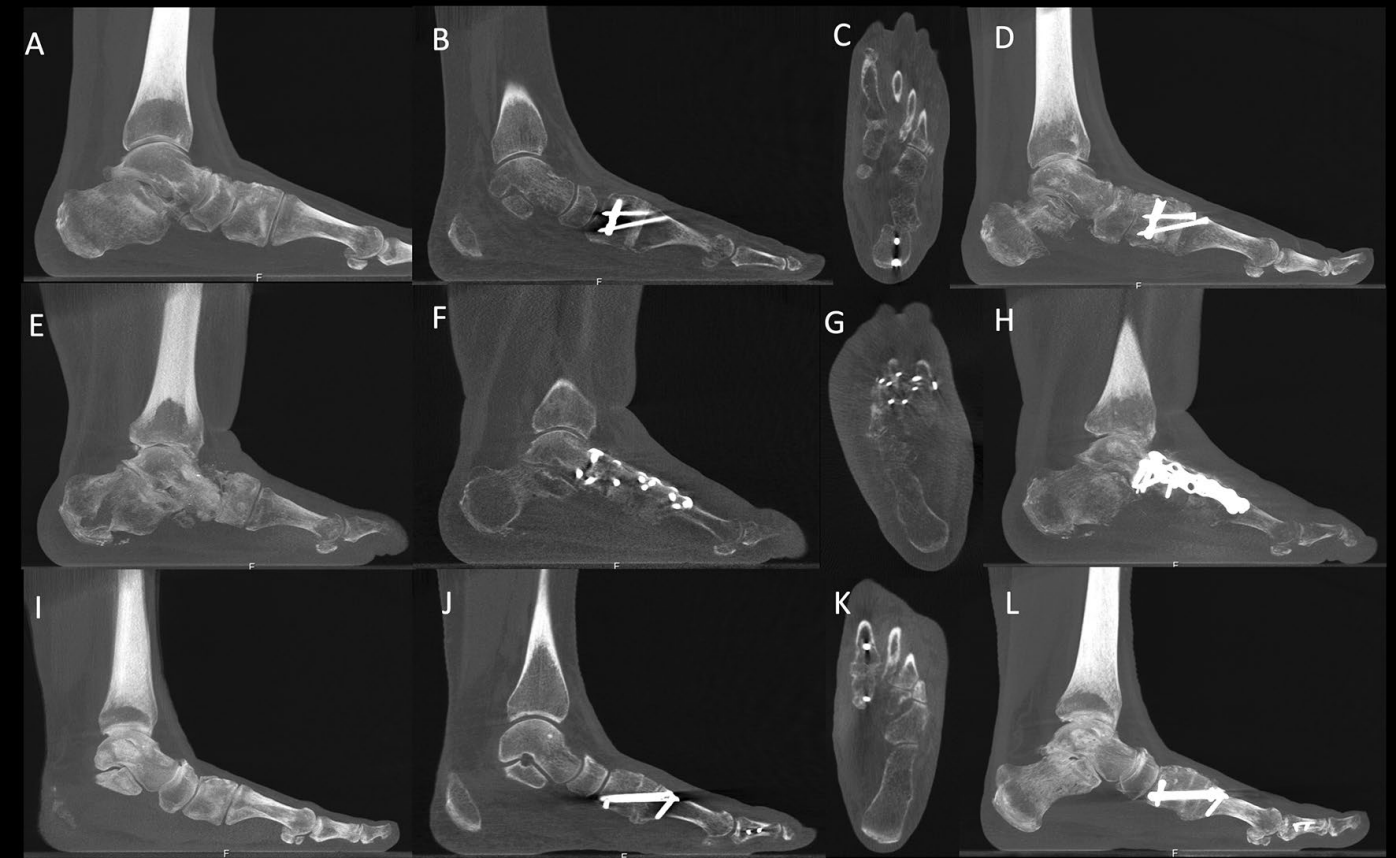
Examples of preoperative and postoperative weight-bearing CT (WBCT) images for the three different types of implants utilized for fixation of the LapiCotton procedure (**A**–**D** medial cuneiform post; **E**–**H** plate and screws; **I**–**L** intramedullary device). First column on the left (**A**, **E** and **I**) shows a thick sagittal cut of the preoperative deformity. Second (**B**, **F** and **J**) and third columns (**C**, **G**, and **K**) demonstrate, respectively, sagittal and axial 3-month postoperative WBCT imaging of the first tarsometatarsal fusion site, with full integration of the allograft and complete bone healing with all three different implants. Last column on the right (**D**, **H** and **L**) shows a thick sagittal WBCT image of the postoperative construct

**Fig. 5:**
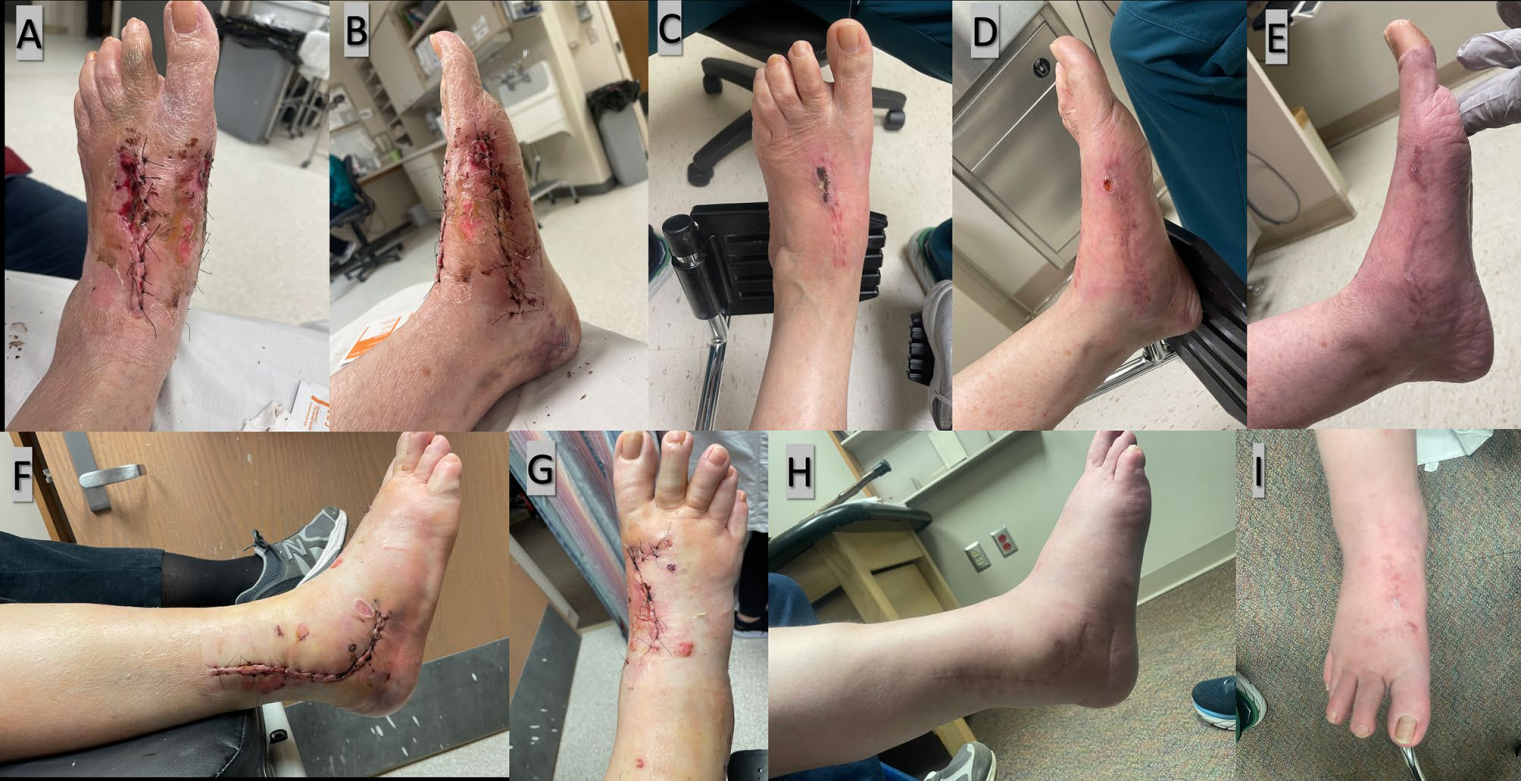
Clinical images of the two patients that presented with minor complications with superficial wound dehiscence. One patient treated for midfoot arthritis had a delayed wound healing at 3 weeks (**A** and **B**) and 6 weeks (**C** and **D**) which was resolved with simple wound care and dressings. The patient was completely healed by the 6th postoperative week (**E**). The second patient underwent LapiCotton procedure for progressive collapsing foot deformity in the setting of a total ankle replacement and had a superficial dehiscence by the 3rd postoperative week (**F** and **G**) that was resolved with conventional wound care and dressings. Patient demonstrated complete healing in the 6-week postoperative visit (**H** and **I**)

A summary of the demographics of all included patients, with diagnosis, comorbidities, allograft wedge sizes utilized and TFMA measurements performed are presented in Table 1. Associated surgical procedures performed concomitantly with LapiCotton are presented in Table 2. Patients had a median follow-up of 5.9 months (range 3–12 months; 95% CI 4.5–7.2). The median allograft wedge size used was 8 mm (range 5–19 mm; mode 8 mm). Regarding the type of implant utilized for LapiCotton fixation, a total of 16 medial cuneiform post implants were used (73%), five plate/screw fixations (23%) and one intramedullary nail (4%) (Fig. 4). Minor complications were observed in two patients (9%), consisting of two superficial dehiscence that resolved after 6 weeks with no need for additional surgical procedures (Fig. 5). Major complications occurred in only one patient (4.5%), who was treated for MA and developed limited ROM of the first MTP joint and mild cock-up deformity of the first toe, as well as a deep dehiscence and deep infection of the dorsal wound utilized for the concomitant fusion of the second and third TMT joints. This required intravenous antibiotics, surgical irrigation and debridement and hardware removal. No other patients reported or were found to have symptoms such as sesamoid pain, limited ROM of the first MTP joint, or lateral column pain.

**Table 1:** Summary of demographics, medial longitudinal arch collapse diagnosis, comorbidities, allograft wedge sizes utilized during the LapiCotton procedure, pre- and postoperative weight-bearing CT sagittal plane talus-first metatarsal angle (TFMA) for the 22 patients included in the study

Patient	Side	Gender	BMI	Age	DM	Smoking	Rheumatoid	Diagnosis	Allograft Size (mm)	Preop TFMA (°)	Postop TFMA (°)
Patient 1	L	M	26.44	70	NO	NO	NO	PCFD	5	20.6	5.9
Patient 2	R	F	39.45	37	NO	NO	YES	PCFD	8	23.9	12.65
Patient 3	R	F	45.42	40	NO	NO	YES	PCFD	8	8.35	7.6
Patient 4	L	F	31.28	45	NO	NO	NO	PCFD	10	13.05	5.1
Patient 5	L	F	31.62	33	NO	NO	NO	PCFD	10	20.85	13.95
Patient 6	L	F	28.29	72	YES	NO	NO	PCFD	8	17.4	9.35
Patient 7	R	F	40.88	19	NO	NO	NO	PCFD	8	3.9	4.1
Patient 8	R	F	30.71	33	NO	NO	NO	PCFD	10	16.9	− 0.15
Patient 9	L	M	36.41	56	NO	NO	NO	PCFD	10	13.8	5.05
Patient 10	L	F	32.07	47	YES	NO	NO	PCFD	5	7.5	3.6
Patient 11	R	F	32	51	NO	NO	NO	PCFD	8	12.75	5.6
Patient 12	L	F	24.98	39	NO	NO	NO	HV	5	10.9	6.45
Patient 13	R	M	26.99	58	NO	NO	NO	HV	5	5.05	1
Patient 14	L	F	31.37	59	NO	NO	NO	HV	5	11.45	4.35
Patient 15	L	F	29.58	61	NO	NO	NO	HV	8	14.75	7.25
Patient 16	L	F	27.77	65	NO	NO	NO	HV	10	6.85	− 5.55
Patient 17	L	F	23.03	75	NO	NO	NO	MA	8	32.85	13.45
Patient 18	R	F	17.99	58	NO	NO	NO	MA	5	21.65	3.9
Patient 19	R	F	56.07	61	NO	NO	NO	MA	19	19.85	− 1.25
Patient 20	R	F	40.9	54	NO	NO	NO	MA	10	14.6	3.8
Patient 21	L	M	37.16	60	NO	NO	NO	MA	12	14.9	6.95
Patient 22	R	M	32.74	65	NO	NO	YES	MA	8	23.7	15.4

*L* left, *R* right, *BMI* body mass index, *PCFD* progressive collapsing foot deformity, *HV* hallux valgus deformity, *MA* midfoot arthritis, *Preop* preoperative, *Postop* postoperative, *mm* millimeters, ^o^ degrees

**Table 2:** Summary of the concomitant surgical procedures performed at the time of the LapiCotton for the 22 included patients in the study

Patient	Diagnosis	Associated procedures
Patient 1	PCFD	MDCO + LCL + PTT reattachment + Strayer
Patient 2	PCFD	MDCO + LCL + spring ligament retensioning + PTT reattachment + PB to PL + Strayer
Patient 3	PCFD	MDCO + PTT reattachment + Strayer
Patient 4	PCFD	MDCO + spring ligament retensioning with STA + deltoid ligament retensioning with STA + PTT reattachment + Strayer
Patient 5	PCFD	MDCO + spring ligament retensioning with STA + deltoid ligament retensioning with STA + FDL transfer + Strayer
Patient 6	PCFD	MDCO + FDL transfer + Strayer
Patient 7	PCFD	MDCO
Patient 8	PCFD	MDCO + FDL reconstruction + Strayer
Patient 9	PCFD	MDCO + PB to PL
Patient 10	PCFD	Total ankle replacement (lateral approach) + Brostrom + MDCO
Patient 11	PCFD	Total ankle replacement (lateral approach) + Brostrom
Patient 12	HV	No
Patient 13	HV	Akin + modified McBride
Patient 14	HV	Akin + modified McBride
Patient 15	HV	Akin + modified McBride
Patient 16	HV	Akin + modified McBride
Patient 17	MA	23 TMT fusion (plate) + Akin + modified McBride
Patient 18	MA	1 NC fusion (plate) + 23 TMT fusion (posts)
Patient 19	MA	123 NC fusions + 23 TMT fusions (plates)
Patient 20	MA	23 TMT fusions (posts)
Patient 21	MA	TN fusion + 123 NC fusions + 23 TMT fusions (plates)
Patient 22	MA	23 TMT fusion (posts) + Akin + modified McBride

*PCFD* progressive collapsing foot deformity, *HV* hallux valgus deformity, *MA* midfoot arthritis, *TAR* total ankle replacement, *MDCO* medial displacement calcaneal osteotomy, *LCL* lateral column lengthening, *PTT* posterior tibial tendon, *PB* peroneus brevis, *PL* peroneus longus, *FDL* flexor digitorum longus, *TMT* tarsometatarsal joint, *NC* naviculocuneiform joint, *TN* talonavicular joint, *1* first, *2* second, *3* third, *STA* synthetic tape augmentation

**Fig. 6:**
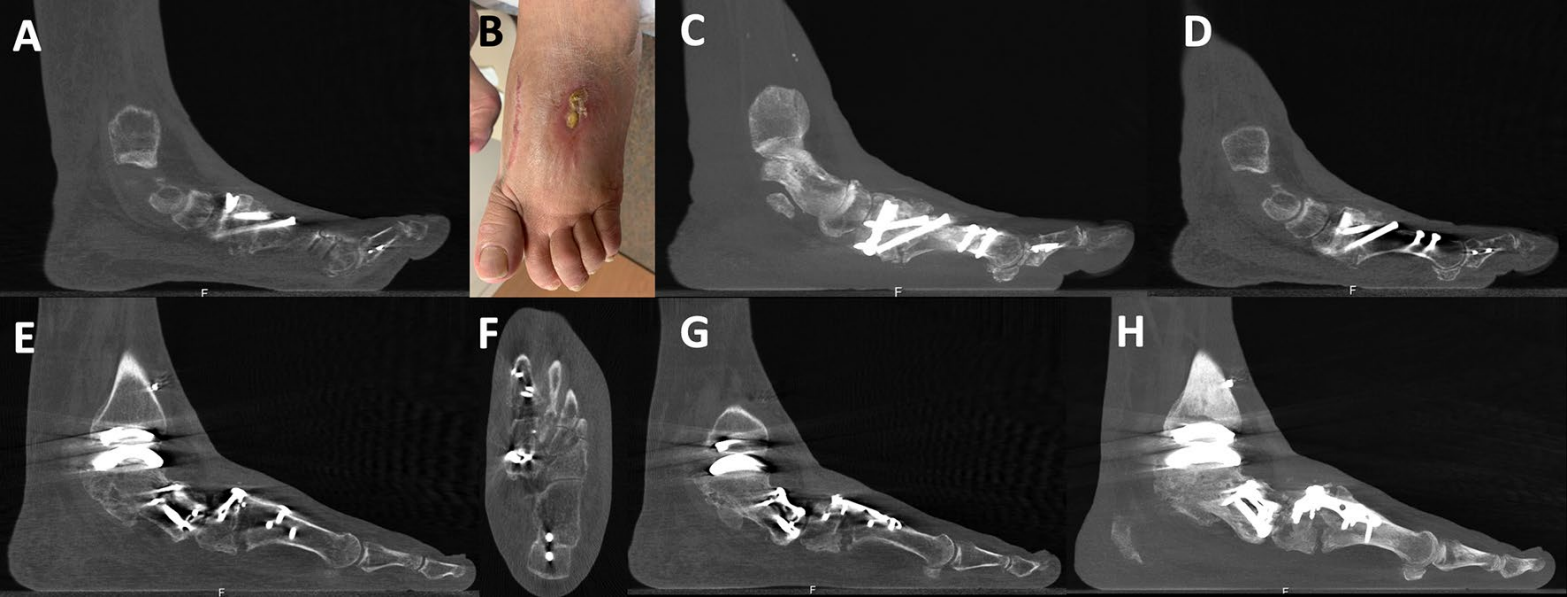
Images displaying the two patients with absence of bone healing at the 3 months weight-bearing CT (WBCT) imaging. Images **A**–**D** exhibit a patient with midfoot arthritis that underwent the LapiCotton as part of the surgical treatment. WBCT sagittal plane image at 12 weeks showing incomplete integration with no complete healing of the fusion interfaces between allograft wedge and hot bone (**A**). The same patient developed a deep dehiscence and infection during further follow-up (**B**) that required reoperation with irrigation and debridement, hardware removal and antibiotic beads placement. The 6-month postoperative WBCT sagittal images depict a non-union of the fusion site, particularly at the distal interface, with partial reabsorption of the allograft wedge and hardware breakage. Images E to H show WBCT images of a patient with progressive collapsing foot deformity treated with a LapiCotton after a total ankle replacement. Sagittal (**E**) and axial (**F**) plane WBCT images at 3-months follow-up demonstrate absence of adequate bone bridging in the first TMT fusion site with partial reabsorption of the allograft wedge. At 6 months, thick (**G**) and single slice (**H**) sagittal WBCT images confirm the non-union of the fusion site. However, the patient is asymptomatic and does not demonstrate progressive collapse of the medial longitudinal arch

Considering healing of the fusion site at the 3-months follow-up time point, 20 patients (91%) had complete healing of both surfaces of the fusion site. Two patients, however (9%), did not demonstrate adequate expected healing (> 50% bone bridging in both surfaces of the fusion site) (Fig. 6). One of the cases was a PCFD patient that received a LapiCotton for a medial arch collapse in the context of a total ankle replacement. Clinically, the patient was doing fine with no pain at the first TMT joint, and no signs of additional progressive collapse or loss of correction. The WBCT was repeated in this patient at the 6-months follow-up. At that point, a radiographic non-union of the fusion site was confirmed, but clinically the patient was still asymptomatic and back to all his previous activities. The second case with no signs of full healing of the LapiCotton fusion site was the same MA patient who developed the deep dehiscence and infection and needed additional intervention with irrigation and debridement, absorbable antibiotic bead placement and hardware removal of the second and third tarsometatarsal fusion hardware. WBCT was repeated at the 6-month follow-up, confirming the presence of radiographic and clinical non-union with loss of correction of the longitudinal arch. This patient is currently under assessment by our soft tissue coverage team for treatment of the dehiscence and will need a revision procedure once the soft tissue coverage problem is solved.

**Fig. 7:**
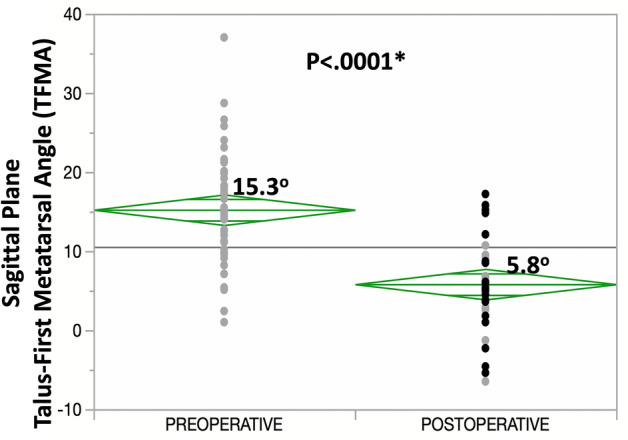
Graphical plot for the comparison of preoperative and postoperative 3-month sagittal plane weight-bearing CT (WBCT) talus-first metatarsal angle (TFMA) in 22 patients treated with the LapiCotton procedure. The mean values are depicted by the horizontal line within each diamond, the 95% confidence interval is indicated by the top and bottom of each diamond, and the mean value for all measurements combined is shown by the long horizontal line

The inter-observer reliability for measurements of sagittal plane TFMA was excellent, with an ICC of 0.86. The average improvement in the collapse of the medial longitudinal arch of the foot, measured by the sagittal plane TFMA was 9.4° (95% CI 6.7–12.1°; *p* < 0.0001), with a mean preoperative angle of 15.3° (95% CI 13.3–17.2°) and a mean postoperative angulation of 5.8° (95% CI 3.9–7.8°) (Fig. 7).

## Discussion

In this study, we report the early results and complication rates of a prospective cohort of patients with medial longitudinal foot arch collapse secondary to either PCFD, HV or MA treated with the LapiCotton procedure. To the authors’ knowledge, this is the first time a series of patients treated with a distraction plantarflexion arthrodesis of the first TMT joint as a primary surgical procedure for collapse of the medial column of the foot is reported in the literature. Because of the considerable incidence of non-unions reported in the literature for the conventional modified Lapidus technique of up to 8% [20], there was an obvious concern in utilizing an allograft wedge in the first TMT fusion site, and expecting healing of two interfaces between host and allograft bone, rather than a single host bone fusion interface. There was also some concern regarding the occurrence of other possible complications related to the lengthening and repositioning of the first ray in the sagittal plane. In the first 22 patients treated with the technique, we found a relatively low rate of early complications, 9% for minor complications (superficial dehiscence), 4.5% for major complications (deep dehiscence/infection) and only 9% absence of full healing of the fusion site (both fusion surfaces) after 3 months.

The use of structural distraction bone graft in the first TMT joint is not an original idea [27, 28]. It has been described to reestablish relative shortening of the first ray in sequela of traumatic Lisfranc joint injuries and as revision salvage procedures for first TMT non-unions [29]. Komenda et al. reported its use in a total of 11 patients of a cohort of 32 subjects with post-traumatic foot deformities [28]. Although detailed results of the 11 patients that underwent the distraction first TMT arthrodesis were not provided, the authors found a 3% rate of non-unions and 21% of complication rate when considering the entire cohort [28]. A good correction of the radiographic alignment of the medial column was also achieved by measuring the talus-first metatarsal angle in lateral conventional radiographic views, with measurements improving on average from 16° of dorsiflexion preoperatively to 6° of dorsiflexion postoperatively [28]. Sangeorzan et al. reported the use of a structural iliac autograft in a nondetermined subset of a total of 16 patients undergoing salvage revision first TMT arthrodesis for patients with Lisfranc joint fractures or fracture dislocations that failed initial surgical treatment [27]. They found an overall 18% non-union rate, absence of major complications, and 69% good to excellent functional results [27]. In contrast, our cohort presented with two patients (9%) that did not demonstrate complete healing after 3 months of surgery. The main difference is that our assessment was performed with WBCT imaging rather than conventional radiographs, increasing the sensitivity for the diagnosis of delayed healing/non-union [49–51]. The rate of radiographic non-unions in these other reported cohorts could potentially be higher if computed tomography (CT) imaging was used for assessment. However, a true comparison is difficult since the diagnosis, indications and fixation options are considerably different. Recently, Barg et al. in a systematic review for hallux valgus surgical treatment reported an overall non-union rate for primary first TMT arthrodesis of 3.8% in a pool of 261 patients from five different studies, with a 95% CI from 1.1 to 7.8% [20]. Compared to this reported data, our 9% incomplete healing after 3 months and 4.5% (single patient) with a true non-union needing surgical intervention could be interpreted as expected and comparable to the available data of traditional modified Lapidus procedures.

The healing rate of allograft bone wedges/bone block in foot and ankle surgery has also been a matter of debate over the last decade. The use of femoral head allograft to treat large bone defects in areas such as the ankle and hindfoot joints has been linked to demonstrate around only 50% of bone healing rate [52, 53]. A recent literature review from Lareau et al. found an 86% probability of fusion when using structural allografts in foot and ankle when compared to a 94% probability when using structural autografts [54]. Hollawel et al. argued for an increased risk of allograft bone block non-union in diabetics and smokers, although no statistical difference was found [55]. When assessing healing of allograft wedges in the setting of lateral column lengthening procedures for PCFD, Foster et al. found a 15% non-union rate (compared to a 7% rate in a group treated with an opening wedge plate) and Grier et al. observed only 6% non-union rate when using allograft wedged and platelet-rich plasma [56, 57]. Burke et al. described a 5% non-union rate on a series of 38 patellar interposition allografts for first metatarsophalangeal distraction arthrodesis [58]. Our non-union rates are comparable to the reports of using allograft wedges for lengthening osteotomies and distraction arthrodesis of small joints such as the first TMT.

Complications of the modified Lapidus first TMT joint arthrodesis are not rare and might reach up to 16% of cases [21, 41]. HV deformity recurrence has been reported from 2 to 13% of the cases, a variable not reported in PCFD and MA studies [40, 41, 59]. Barg et al. in a systematic review for HV surgical treatment reported a 6.6% (95% CI 3.9–9.9%) reoperation rate and 11.4% infection rate (95% CI 0.3–35%) with the use of the modified Lapidus. Our complication rate was comparable to that previously reported in the literature. We had 9% rate of minor complications with superficial dehiscence and only 4.5% of major complications with one patient presenting with deep dehiscence, deep infection and non-union after 6 months, needing reoperation. Interestingly, no complaints about new metatarsalgia or sesamoid pain were noted, a concern when changing the relative length and position of the metatarsals.

The importance of the first ray in restoring the mechanical function of foot tripod has been highlighted in the literature [3, 10, 16, 30, 60–62]. An unstable medial column has been linked to the pathogenesis of PCFD, HV, and MA [8, 9, 63]. The substantial structural importance of the first ray and its long lever arm during gait for the midfoot, hindfoot, and, mainly, the ankle joints may play a key feature in the development and progression of these diseases [64–67]. The challenges of preserving first ray length and avoiding first ray dorsiflexion when performing a conventional modified Lapidus first TMT joint fusion are considerable. The results of shortening and absence of plantarflexion might hinder the mechanical advantage that the first ray could play in correcting medial longitudinal arch collapse in patients with PCFD, HV and MA, which could be linked to recurrence and progression of the deformities [10, 32]. An expected loss in first metatarsal length of up to 4.1 mm has been described in clinical studies and up to 8.1 mm shortening has been reported in cadaveric studies [25, 26, 68]. Until recently, shortening and relatively dorsiflexed position of the first ray in the setting of first TMT joint arthrodesis had not been associated with poorer clinical outcomes [25, 69]. However, recently Nishikawa et al. reported a significant postoperative relative shortening of the first ray of 2.3 mm on average, and demonstrated that the clinical functional outcomes, measured by the Lower Extremity Functional Scale, significantly decreased as the relative shortening of the first metatarsal increased (*p* < 0.05) [70]. Nishikawa et al. also later reported the presence of relative postoperative dorsiflexion of the first metatarsal following conventional modified Lapidus procedure (average 2.5° of dorsiflexion) in 78% of a total of 36 HV patients. They also found a significant correlation between first metatarsal shortening and poorer functional outcomes (SF-12 and Lower Extremity Functional Scale) [24]. Our understanding is that the results of these reported studies highlight the importance of improving or at least preserving the length and sagittal plane position of the first metatarsal in patients with collapse of the medial longitudinal arch, advocating in favor of the LapiCotton procedure. Busch et al. was also able to demonstrate a significant correlation between increased first ray dorsiflexion (lateral talus-first metatarsal angle) and the occurrence of postoperative transfer metatarsalgia [23]. In our study, we found a significant improvement in the TFMA of around 9.4 degrees consistent with improvement in the sagittal plane position of the first ray. Our understanding is that this substantial correction of the sagittal plane position of the first ray in plantarflexion by means of the LapiCotton procedure can help to reestablish the foot tripod, potentially positively influencing improved outcomes in the long term for patients with collapse of the medial longitudinal arch of the foot. Future continued assessment of the patients that underwent the procedure will be necessary to support this possibility. The advent of three-dimensional measurements, distance and coverage maps may help in evaluating proper correction obtained by the LapiCotton in further studies [71–75].

This study has several limitations. First and foremost, even though this was a prospective cohort study, there was no control group or comparison with other surgical procedures such as a conventional modified Lapidus dirst TMT joint arthrodesis, and the follow-up is short. However, since we report on the results of a relatively novel technique that includes the addition of a dorsal opening wedge allograft in the first TMT fusion site, we judged it was important to report early radiographic results, complication, and healing rates. Also, no clinical patient-reported outcomes were assessed in this study. It is our intention to assess and report these outcomes in longer-term follow-up studies. Another important limitation was that we assessed the use of the LapiCotton procedure for patients with different causes for medial column collapse (PCFD, HV and MA). An assessment for each one of the diagnoses in isolation will be important in the future, since usually, the complexity of some cases, particularly severe MA and PCFD cases, can negatively influence the incidence of complications and non-union rate. The fact that multiple associated procedures were performed in combination with the LapiCotton in most of our patients also increases the difficulty in the understanding and interpretation of radiographic and complication/healing rates. However, the reality is that very frequently more than one procedure is necessary to treat those patients. The fact that all procedures were performed by a single surgeon validates the homogeneity of the surgical technique and perioperative protocol, however**,** hinders the generalization and reproducibility of the results. Finally, no sample calculation or power analysis was executed; however, the sample size was large enough to demonstrate significant and pronounced improvements in the collapse of the longitudinal arch of the foot, measured by the TFMA.

## Conclusion

In this prospective cohort study of 22 patients treated with the LapiCotton procedure as part of the surgical technique for treatment of pathologies that involve a collapse of the medial column of the foot, we observed a significant amount of postoperative correction of the longitudinal arch collapse deformity, with an average correction of the sagittal plane talus-first metatarsal angle of approximately 10 degrees, and a high healing rate (91%) of the fusion site after 3 months. One clinically stable radiographic non-union (4.5%) and one unstable non-union (4.5%) needing surgical intervention were noted. We also observed a relatively low rate of complications, with two minor complications (9%) and one major complication (4.5%) with deep infection needing surgical treatment in the same patient with the unstable non-union. Our results demonstrate promising initial results for LapiCotton technique in treating collapse of the medial longitudinal arch in patients with PCFD, MA and HV deformities. However, long-term studies are needed.

## Data Availability

According to the ICMJE data sharing police, core records will be shared through Mendeley Data and are available upon request.
